# Cortical degeneration in chronic traumatic encephalopathy and Alzheimer’s disease neuropathologic change

**DOI:** 10.1007/s10072-018-3686-6

**Published:** 2018-12-18

**Authors:** Richard A. Armstrong, Ann C. McKee, Thor D. Stein, Victor E. Alvarez, Nigel J. Cairns

**Affiliations:** 1 Vision Sciences, Aston University, Birmingham, B4 7ET UK; 2 VA Boston HealthCare System, Boston, MA 02130 USA; 3 Department of Neurology, Boston University School of Medicine, Boston, MA 02118 USA; 4 Department of Pathology & Laboratory Medicine, Boston University School of Medicine, Boston, MA 02118 USA; 5 Department of Veterans Affairs Medical Center, Bedford, MA 01730 USA; 6 Departments of Neurology and Pathology & Immunology, Washington University School of Medicine, Saint Louis, MO 63110 USA

**Keywords:** Chronic traumatic encephalopathy (CTE), Alzheimer’s disease neuropathologic change (ADNC), Tauopathy, Laminar distribution

## Abstract

**Objectives:**

An observational study to compare the laminar distributions in frontal and temporal cortex of the tau-immunoreactive pathologies in chronic traumatic encephalopathy (CTE) and Alzheimer’s disease neuropathologic change (ADNC).

**Patients:**

Post-mortem material of (1) four cases of CTE without ADNC, (2) seven cases of CTE with ADNC (CTE/ADNC), and (3) seven cases of ADNC alone.

**Results:**

In CTE and CTE/ADNC, neurofibrillary tangles (NFT), neuropil threads (NT), and dot-like grains (DLG) were distributed either in upper cortex or across all layers. Low densities of astrocytic tangles (AT) and abnormally enlarged neurons (EN) were not localized to any specific layer. Surviving neurons exhibited peaks of density in both upper and lower cortex, and vacuole density was greatest in superficial layers. In ADNC, neuritic plaques (NP) were more frequent, AT rare, NFT and NT were more widely distributed, NT affected lower layers more frequently, and surviving neurons were less frequently bimodal than in CTE and CTE/ADNC.

**Conclusion:**

Tau pathology in CTE and CTE/ADNC consistently affected the upper cortex but was more widely distributed in ADNC. The presence of CTE may encourage the development of ADNC pathology later in the course of the disease.

## Introduction

Chronic traumatic encephalopathy (CTE) is a neurodegenerative disorder associated with repetitive traumatic brain injury (TBI) [1]. Clinical symptoms include impairment of memory and executive function, behavioral change, and motor dysfunction [2]. Neuropathologically, there is reduced gray matter volume affecting the frontal and anterior temporal lobes [3, 4]. A spectrum of tau-immunoreactive pathology is present including neurofibrillary tangles (NFT) and neuropil threads (NT) [3], dot-like grains (DLG) [5], and astrocytic tangles (AT) [2, 6]. Low densities of abnormally enlarged neurons (EN) and vacuolation are also present [5]. By contrast, Alzheimer’s disease neuropathologic change (ADNC) is characterized by the presence of β-amyloid (Aβ) deposits in the form of neuritic plaques (NP) and tau-immunoreactive NFT without an extensive glial pathology [7, 8]. ADNC often occurs as a co-pathology with CTE but the relationship between CTE and ADNC remains controversial [7, 8]. To compare the two pathologies, an observational study of the changes in density of the NFT, NT, DLG, and AT together with EN, surviving neurons, and vacuoles was carried out across cortical regions in CTE alone, CTE with ADNC (CTE/ADNC), and ADNC alone.

## Materials and methods

### Cases

Cases were obtained from Boston University’s CTE center (VA-BU-CLF Brain Bank) (Table 1). With the exception of a boxer for 26 years (case A), all CTE cases had played American football (career durations 11–21 years). Patients had suffered at least one traumatic episode resulting in concussion, some with accompanying loss of consciousness, the majority experiencing multiple traumas. CTE diagnosis was according to criteria of McKee et al. [9] and cases were divided into two groups: (1) four cases of CTE without ADNC and (2) seven cases of CTE with ADNC (CTE/ADNC) with varying densities of β-amyloid (Aβ) deposits and NP [10] (CTE/ADNC). Seven cases of ADNC with no history of traumatic brain injury were also studied, six having a clinical diagnosis of Alzheimer’s disease (AD) and one of dementia/possible AD.

**Table 1:** Demographic features and neuropathology features, including National Institute on Aging-Alzheimer’s association (NIA-AA) guidelines “ABC” scores, of the cases studied. Chronic traumatic encephalopathy (CTE) cases also include frequency of traumatic incidents (first figure frequency of reported concussions, second figure concussions resulting in loss of consciousness), and sporting career length

Case	Sex	Age*	Duration of dementia (years)	Trauma (years)	Career length (years)	A	B	C
CTE								
A	M	60	6	1/1	26	0	1	0
B	M	70	8	3/0	21	0	1	0
C	M	70	11	50/1	17	0	2	0
D	M	70	26	Fr	20	0	2	0
CTE/ADNC								
E	M	75	10	10/2	18	3	2	1
F	M	70	4	10/1	11	1	2	0
G	M	65	10	na	19	2	2	1
H	M	80	15	F	12	2	2	0
I	M	80	40	na	18	3	2	1
J	M	70	9	10/1	19	2	1	1
K	M	60	8	25/1	21	3	2	0
ADNC								
A	F	90	5	–	–	3	2	1
B	M	70	3	–	–	2	2	1
C	F	80	5	–	–	2	1	0
D	F	85	12	–	–	3	2	1
E	F	90	9	–	–	2	1	1
F	M	70	14	–	–	1	2	1
G	M	70	8	–	–	3	2	1

*ADNC* Alzheimer’s disease neuropathologic change, *M* male, *F* female, *Fr* frequent, *Age rounded to nearest 5-year age interval to protect subject identities, *na* not available

### Tissue preparation

The local Institute Review Board of Boston University approved the study and were carried out according to the 1995 Declaration of Helsinki (as modified in Edinburgh, 2000). Brains were fixed in 10% neutral buffered formalin for at least 2 weeks, paraffin-embedded, and sections cut at 6 μm. Blocks were taken from (1) frontal lobe to study the superior frontal gyrus (SFG) (BA 8,6), (2) temporal pole (BA 38,36), (3) temporal lobe to study the superior temporal gyrus (STG) (BA 22), and (4) medial temporal lobe to study the entorhinal cortex (EC) (BA 28). Immunohistochemistry was performed using antibodies against phosphorylated tau (AT8, Pierce Endogen, Rockford, IL, USA; 1:2000). Each slide was scanned and subsequently visualized on a PC using Aperio Image-Scope Software (Leica Biosystems Inc. Buffalo Grove, IL, USA).

### Morphometric methods

The laminar distribution the histological features was studied using previously described methods [11]. Two traverses of 250 × 50-μm fields arranged contiguously and superimposed over the image from pia mater to white matter were located in each region, one along the upper side of the gyrus adjacent to the crest and the second at the deepest point of the sulcus. NFT, NT, DLG, NP, AT, EN, surviving neurons, and vacuoles were counted within each field along the traverse.

### Data analysis

Changes in density of a histological feature with distance across the cortex were analyzed using a polynomial curve-fitting procedure to establish the curve of best fit (STATISTICA software, Statsoft Inc., 2300 East 14th St, Tulsa, OK, 74104, USA) [12]. Chi-square (*χ*^2^) contingency table tests were used to determine differences in cortical distribution among the three groups of cases.

## Results

**Fig. 1:**
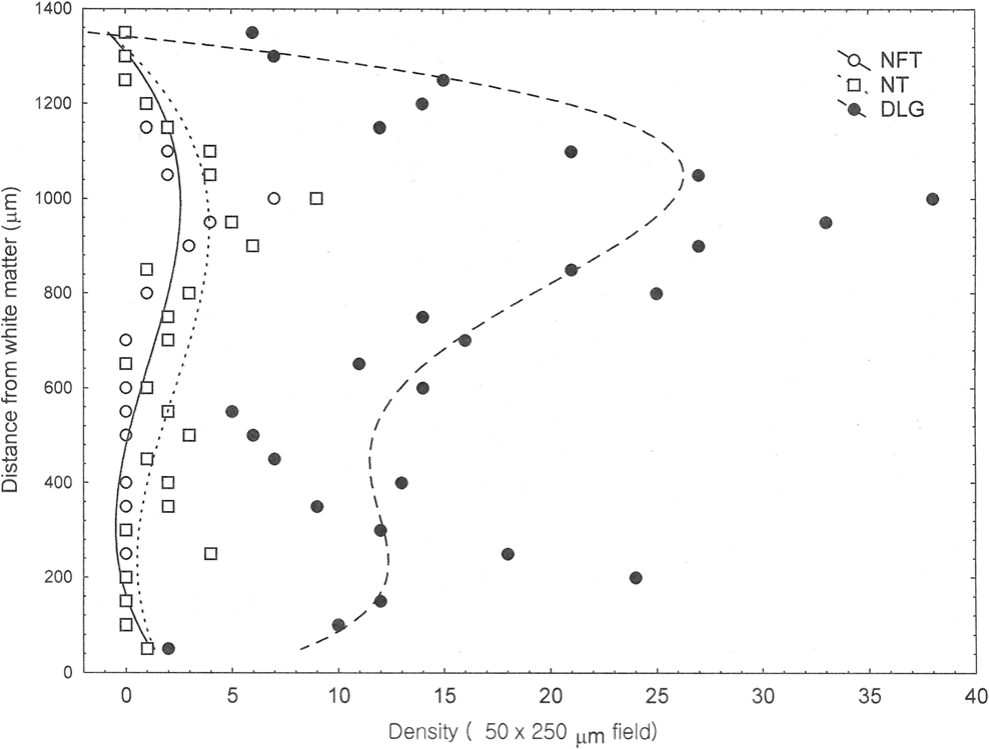
Quantitative analysis of the laminar distribution of the tau-immunoreactive pathology at the temporal pole (TP) in a case of chronic traumatic encephalopathy (CTE) with Alzheimer disease neuropathologic change (ADNC). Curves of best fit: NFT: third-order polynomial (*r* = 0.70, *P* < 0.01), NT: third-order polynomial (*r* = 0.63, *P* < 0.01), DLG: fourth-order polynomial (*r* = 0.75, *P* < 0.01)

The curve fitting procedure is illustrated in Fig. 1 (Case F, CTE/ADNC, TP). The distributions of the NFT (*r* = 0.70, *P* < 0.01) and NT (*r* = 0.63, *P* < 0.01) were fitted by third-order polynomial curves with peaks of maximum density in the upper cortex. The distribution of the DLG was fitted by a fourth-order polynomial (*r* = 0.75, *P* < 0.01) suggesting a bimodal distribution with density peaks in upper and lower cortex, the upper peak being larger than the lower.

In the data as a whole (Table 2), AT were present largely in CTE and CTE/ADNC scattered across all layers. Conversely, NP were largely present in ADNC most frequently in the upper cortex. The distributions of the NFT (*χ*^2^ = 3.86, *P* > 0.05), NT (*χ*^2^ = 3.72, *P* > 0.05), and DLG (*χ*^2^ = 10.67, *P* > 0.05) were not significantly different in CTE and CTE/ADNC. However, there were differences in the NFT (*χ*^2^ = 15.34, *P* < 0.01), NT (*χ*^2^ = 28.06, *P* < 0.001), and DLG (*χ*^2^ = 12.15, *P* < 0.05) in CTE and CTE/ADNC compared with ADNC. In CTE and CTE/ADNC, the NFT, NT, and DLG were confined to the upper layers more frequently and NT to the lower layers less frequently than in ADNC. The surviving neurons were also more frequently bimodal in CTE and CTE/ADNC than ADNC. There were no differences in distribution of the EN or vacuolation in CTE, CTE/ADNC, and ADNC. Laminar changes were similar in gyri compared with the sulci and in case A (the boxer) compared with the other CTE cases representing ex footballers.

**Table 2:** Comparison of the frequencies of the tau-immunoreactive pathology (*NFT* neurofibrillary tangles, *NT* neuropil threads, *DLG* dot-like grains, *AT* astrocytic tangles, *EN* enlarged neurons, *SN* surviving neurons, *Vac* vacuolation) in chronic traumatic encephalopathy (CTE) cases with and without Alzheimer disease neuropathologic change (ADNC) and in ADNC alone. (*U* upper cortex, *M* middle layers of cortex, *L* lower cortex, *NS* no significant change across cortex)

				Laminar distribution			
Cases	Pathology	*U*	*L*	*U* > *L*	*U* = *L*	*U* < *L*	NS
CTE	NFT	8	0	7	1	0	14
	NT	13	3	4	1	0	12
	DLG	19	3	7	0	0	2
	AT	3	1	0	0	0	5
	EN	0	4	0	0	0	23
	SN	7	0	10	3	1	10
	Vac	16	0	2	4	2	8
	NP	0	0	0	0	0	0
CTE/ADNC	NFT	20	1	5	2	0	26
	NT	19	2	7	2	3	14
	DLG	25	0	16	5	1	7
	AT	1	2	1	1	0	21
	EN	2	10	0	2	0	38
	SN	18	2	11	9	0	16
	Vac	30	5	11	3	0	6
	NP	0	0	0	0	0	6
ADNC	NFT	4	4	7	3	0	35
	NT	4	17	5	0	4	11
	DLG	10	2	21	5	2	4
	AT	0	1	2	0	1	0
	EN	2	17	0	1	1	29
	SN	19	1	3	5	1	24
	Vac	32	3	6	1	0	11
	NP	13	5	5	0	0	4

Comparison of frequencies (*χ*^2^ contingency tables). CTE compared with CTE/ADNC: NFT *χ*^2^ = 3.86 (4DF, *P* > 0.05), NT *χ*^2^ = 3.70 (5DF, *P* > 0.05), DLG *χ*^2^ = 10.67 (5DF, *P* > 0.05); AT *χ*^2^ = 6.44 (4DF, *P* > 0.05), EN *χ*^2^ = 2.61 (3DF, *P* > 0.05), SN *χ*^2^ = 5.55 (5DF, *P* > 0.05), Vac *χ*^2^ = 12.72 (5DF, *P* < 0.05); Totals for CTE compared with ADNC: NFT *χ*^2^ = 15.34 (4DF, *P* < 0.01), NT *χ*^2^ = 28.06 (5DF, *P* < 0.001), DLG *χ*^2^ = 12.15 (5DF, *P* < 0.05), AT *χ*^2^ = 18.70 (5DF, *P* > 0.01), EN *χ*^2^ = 1.41 (4DF, *P* > 0.05), SN *χ*^2^ = 16.12 (5DF, *P* < 0.01), Vac *χ*^2^ = 4.45 (6DF, *P* > 0.05)

## Discussion

The data suggest the tau pathology of frontal and temporal lobes in CTE and CTE/ADNC affected upper cortical layers typical of the tauopathies [12, 13]. In addition, in some regions, the distribution of the tau pathology affected either both upper and lower cortex, or a more uniform distribution was present.

The distribution of the surviving neurons in CTE and CTE/ADNC was highly variable. In normal elderly brain, larger neuronal perikarya in frontal and temporal cortex peak in upper and lower cortex, the upper peak being larger than the lower. In CTE and CTE/ADNC, a similar distribution was observed in 21/87 regions, and in a further 12 regions, the densities were similar in upper and lower cortex suggesting loss of neurons in upper layers. In 25 regions, a single density peak was present in upper cortex suggesting loss of lower neurons and in a further 26 regions, there were no significant changes in neuronal density across the cortex indicating a more general loss of neurons across the cortex. Where a more localized distribution of EN was present, maximum density occurred in the lower layers similar to other tauopathies [12–14]. Vacuolation of the superficial layers was also a consistent finding in CTE and CTE/ADNC and in many gyri, the density of vacuoles decreased across the cortex.

Difference in the distribution of the pathology in CTE and CTE/ADNC compared with ADNC [14], suggested the tau pathology of CTE/ADNC was more restricted to the upper layers compared with ADNC. If CTE is the primary pathology, related to brain trauma, ADNC tau pathology may develop later in the disease remaining more or less confined to the upper cortical layers. If ADNC is the primary pathology, the tau pathology spreads earlier to affect more of the cortical layers than in CTE/ADNC. ADNC cases were older than the CTE and CTE/ADNC cases, a factor which could also have affected the laminar distributions in ADNC. ADNC cases also had a mixture of males and females unlike the CTE cases, which were exclusively male, but no differences have been observed in laminar distribution between males and females in several disorders [12, 13, 15]. The data also suggest that ADNC develops later in CTE, consistent with the hypothesis that repetitive brain injury also encourages the development of ADNC.

In conclusion, pathological changes in frontal and temporal lobes in CTE and CTE/ADNC most consistently affect upper cortical layers typical of the tauopathies. Lower layers can also be affected but usually to a lesser extent. In addition, there are differences in laminar distributions of the tau pathology in CTE with or without ADNC and ADNC alone consistent with the later development of ADNC in a subset of CTE cases.

## References

[1] Jordan BD (2013). The clinical spectrum of sport-related traumatic brain injury. Nat Rev Neural.

[2] Saing T, Dick M, Nelson PT, Kim RC, Cribbs DH, Head E (2012). Frontal cortex neuropathology in dementia pugilistica. J Neurotrauma.

[3] McKee AC, Stein TD, Nowinski CJ, Stern RA, Daneshvar DH, Alvarez VE, Lee HS, Hall G, Wojtowicz SM, Baugh CM, Riley DO, Kubilus CA, Cormier KA, Jacobs MA, Martin BR, Abraham CR, Ikezu T, Reichard RR, Wolozin BL, Budson AE, Goldstein LE, Kowall NW, Cantu RC (2013). The spectrum of disease in chronic traumatic encephalopathy. Brain.

[4] McKee AC, Daneshvar DH, Alvarez VE, Stein TD (2014). The neuropathology of sport. Acta Neuropathol.

[5] Armstrong RA, McKee AC, Stein TD, Alvarez VE, Cairns NJ (2016). A quantitative study of tau pathology in 11 cases of chronic traumatic encephalopathy. Neuropathol Appl Neurobiol.

[6] Stein TD, Alvarez VE, McKee AC (2014). Chronic traumatic encephalopathy: a spectrum of neuropathological changes following repetitive brain trauma in athletes and military personnel. Alzheimers Res Ther.

[7] Graham DI, Gentleman SM, Lynch A, Roberts GW (1995). Distribution of beta-amyloid protein in the brain following severe head-injury. Neuropath Appl Neurobiol.

[8] Stein TD, Montinegro PH, Alvarez VE, Xia W, Crary JF, Tripodis Y, Daneshvar DH, Mez J, Soloman T, Meng G, Kubilus CA, Cormier KA, Meng KA, Babcock K, Kiernan P, Murphy L, Nowiski CK, Martin B, Dixon D, Stern RA, Cantu RC, Kowall NW, McKee AC (2015). Beta-amyloid deposition in chronic traumatic encephalopathy. Acta Neuropathol.

[9] McKee AC, Cairns NJ, Dickson DW, Folkerth RD, Keene CD, Litvan I, Perl D, Stein TD, Vonsattel JP, Stewart W, Tripodis Y, Crary JF, Bienick KF, Dams-O’Connor K, Alverez VF, Gordon WA, the TBI/CTE group (2016). The first NINDS/NIBIB consensus meeting to define neuropathological criteria for the diagnosis of chronic traumatic encephalopathy. Acta Neuropathol.

[10] Montine TJ, Phelps CH, Beach TG, Bigio EH, Cairns NJ, Dickson DW, Duyckaerts C, Frosch MP, Masliah E, Mirra SS, Nelson PT, Schneider JA, Thal DR, Trojanowski JQ, Vinters HV, Hyman BT, National Institute on Aging; Alzheimer’s Association (2012). National Institute on Aging-Alzheimer’s Association guidelines for the neuropathologic assessment of Alzheimer’s disease: a practical approach. Acta Neuropathol.

[11] Duyckaerts C, Hauw JJ, Bastenaire F, Piette F, Poulain C, Rainsard V, Javoy-Agid F, Berthaux P (1986). Laminar distribution of neocortical senile plaques in senile dementia of the Alzheimer type. Acta Neuropathol.

[12] Armstrong RA, Cairns NJ (2009). Laminar distribution of the pathological changes in frontal and temporal cortex in eight patients with progressive supranuclear palsy. Clin Neuropathol.

[13] Armstrong RA, Lantos PL, Cairns NJ (2000). Laminar distribution of ballooned neurons and tau positive neurons with inclusions in patients with corticobasal degeneration. Neurosci Res Commun.

[14] Hof PR, Bouras C, Buee L, Delacourte A, Perl DP, Morrison JH (1992). Differential distribution of neurofibrillary tangles in the cerebral cortex of dementia pugilistica and Alzheimer’s disease cases. Acta Neuropathol.

[15] Armstrong RA (2017). Laminar distribution of frontal and temporal cortex in Parkinson’s disease dementia. Neurol Sci.

